# Autonomous pick-and-place using the dVRK

**DOI:** 10.1007/s11548-021-02397-y

**Published:** 2021-05-15

**Authors:** Claudia D’Ettorre, Agostino Stilli, George Dwyer, Maxine Tran, Danail Stoyanov

**Affiliations:** 1 Department of Computer Science, Wellcome/EPSRC Centre for International and Surgical Sciences (WEISS), University College London, London, W1W 7EJ UK; 2 Division of Surgery and Interventional Science, Department of Nanotechnology, University College London, Royal Free Hospital, London, NW3 2QG UK

**Keywords:** Computer-assisted interventions, Robotic surgery, Motion planning, Surgical automation

## Abstract

**Purpose:**

Robotic-assisted partial nephrectomy (RAPN) is a tissue-preserving approach to treating renal cancer, where ultrasound (US) imaging is used for intra-operative identification of tumour margins and localisation of blood vessels. With the da Vinci Surgical System (Sunnyvale, CA), the US probe is inserted through an auxiliary access port, grasped by the robotic tool and moved over the surface of the kidney. Images from US probe are displayed separately to the surgical site video within the surgical console leaving the surgeon to interpret and co-registers information which is challenging and complicates the procedural workflow.

**Methods:**

We introduce a novel software architecture to support a hardware soft robotic rail designed to automate intra-operative US acquisition. As a preliminary step towards complete task automation, we automatically grasp the rail and position it on the tissue surface so that the surgeon is then able to manipulate manually the US probe along it.

**Results:**

A preliminary clinical study, involving five surgeons, was carried out to evaluate the potential performance of the system. Results indicate that the proposed semi-autonomous approach reduced the time needed to complete a US scan compared to manual tele-operation.

**Conclusion:**

Procedural automation can be an important workflow enhancement functionality in future robotic surgery systems. We have shown a preliminary study on semi-autonomous US imaging, and this could support more efficient data acquisition.

## Introduction

Developments in robot-assisted laparoscopic surgery have enabled highly dexterous instrument manipulation with enhanced ergonomics which facilitate precise movement within the anatomy without direct access to the surgical site. This has led to significant growth in robotic surgery as an alternative to traditional laparoscopic surgery [1]. Yet despite the increased uptake of surgical robotics, automation is not currently available in clinical practice due to significant technical difficulties in robust robot perception and control within soft-tissue anatomical areas, and also regulatory considerations [2].

**Fig. 1:**
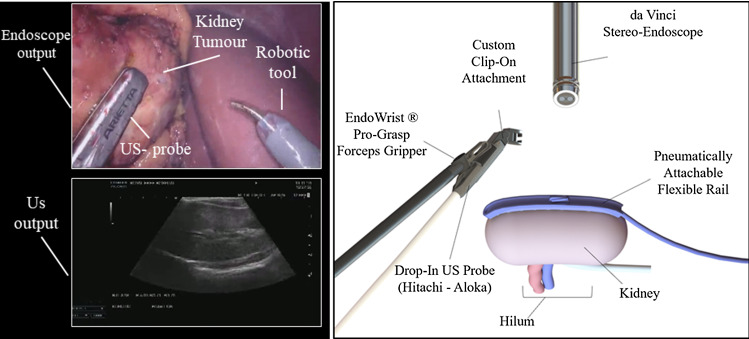
Left: representation of the visual output during RAPN. Surgeons visualise the endoscope output on top and the US image on the bottom. They mentally compute the registration between the two images. Right: model representation of the system design. The suction rail is placed on the kidney surface, and the model of US probe is equipped with an adaptor to slide it along the rail

Robotic-assisted partial nephrectomy (RAPN) is one of the most common robotic-assisted surgical procedures [1]. Removing tumours while retaining healthy tissue has been shown to maximise the patient’s post-operative kidney functions [3]. Intra-operative US imaging in RAPN supports healthy tissue preservation, but manual control of the US probe significantly complicates surgical workflow [4]. As shown in Fig. 1, the endoscopic view of the surgical site and the US image are shown in the surgical console without any co-registration, and the surgeon must interpret multi-modal information and retain it after US probe manipulation for clinical decisions.

Computer-assisted interventions (CAIs) in RAPN have focused on enhancing surgical navigation and improving the management of US imaging probes or the fusion of endoscopic, US and CT modality data. This has been approached from both the image processing and understanding perspective where US information can be used to infer information such as tissue deformation [5] and from an information registration angle co-registering multiple modalities [6]. Hardware solutions have also been proposed to guarantee repeatable grasping of the US transducer by augmenting the probe design [7, 8] and subsequently detecting vessels in the US image and registering to pre-operative CT data. Minor probe modifications have been used to add fiducial markers for automatic detection in the endoscopic image and estimation of the US pose for image overlay [9], although this work does not consider the renal cortex’s curvature. Autonomous US scanning for tumour identification has also been reported [10] that considers the curvature of the tissue’s surface and possible physiological motion within it, approximated with a periodic model. More recently, a similar approach has been extended to work under free-form motion where the US scanning trajectory is manually defined and continuously updated to follow intra-operative tissue motion [11]. Most CAI approaches so far have been relying on the current clinical protocol for intraoperative US scanning where the laparoscopic tool freely moves over the scanning surface.

In this paper, we present a new framework for automated localisation and placement of a pneumatically attachable flexible rail (PAF) [12, 13] using the da Vinci Research Kit (dVRK). This is an incremental but novel step towards assisted US imaging during RAPN to advance surgical workflow beyond manual US probe management.

More specifically, the paper proposes a new platform architecture, algorithm and a pre-clinical user study with five surgeons. This has the following specific contributions:

- Trajectory generation using dynamic time warping motion planning;
- A control scheme based on visual servoing using endoscopic images during the pick-and-place of the rail;
- A comparison between two different surface registration techniques applied to ex vivo porcine kidneys [14];
- Pre-clinical study of system performance with five surgeons comparing semi-autonomous and manual execution of the same task.

Albeit a preliminary study, we investigate and compare the behaviour of expert surgeons and novices in their use of the device and their experience of the algorithm and workflow for automation.

**Fig. 2:**
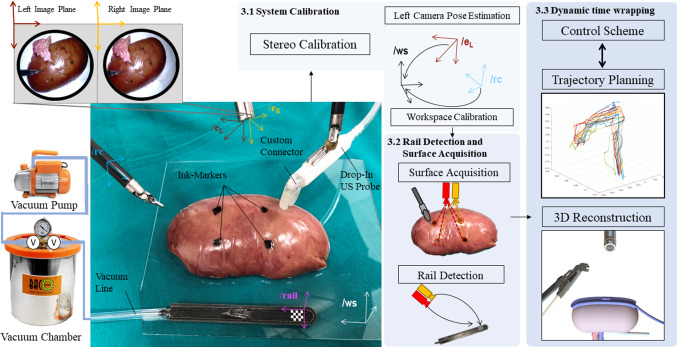
System pipeline from the platform and the methodology point of view. On the bottom left part is shown ex vivo porcine kidney, with the two robotic tools, the stereo endoscope, the black sucker rail and the drop-in US probe. Four ink markers used for surface registration are highlighted on the kidney surface. Orthogonal clockwise reference frame systems are defined by “*/*”. The top left of the figure shows two frames from the left and right camera of the stereo endoscope. On the right side, the ROS node architecture is summarised according to the different methodologies described in the labelled sections

## System overview

### Platform configuration

The dVRK system was used as the surgical robot underpinning our experiments. One of the patient side manipulators (PSM1) is equipped with a large needle driver (LND), while the other PSM2 holds the Pro-Grasp tool. Figure 2 shows the set-up overview alongside the PAF rail, the two PSMs, the stereo endoscope and the porcine kidney. The black rail, presented by the authors in previous works [12, 15], is attached to the kidney surface using a series of bio-inspired vacuum suckers. It is used as a guide on which the surgeon engages and slides the drop-in US probe (3D printed model) to identify vessels and resection margins.

### Software configuration

A custom ROS architecture was developed embedding different software algorithms, shown on the right part of Fig. 2. To enhance the accuracy of the system, a calibration process was performed as a first step (described in “System calibration” Section). The robot calibration involves making minor adjustments to kinematic model parameters to account for factors like manufacturing tolerances, to increase model accuracy. The PSMs are characterised by set-up joints and active joints. It is worth noticing that the instrument’s tip accuracy is generally more sensitive to small angular errors in the base joints than in the more distal ones. Considering this, an approach similar to [14] was followed, attaching the base coordinate frame at the beginning of the active joints. The workspace calibration is defined by the transformation between the workspace and the arm’s remote centre of motion. A vision node (“Rail detection and surface acquisition for kidney registration” Section) was necessary to deal with the rail tracking and the kidney surface registration. The control scheme node (“Dynamic time warping trajectory planning and control features” Section) was introduced to accomplish safety and accuracy requirements.

## Methods

### Notation

Scalars are represented by plain letters, e.g. $\lambda$, vectors by bold symbols, e.g. $\mathbf {x}$. Orthogonal clockwise reference frames are defined with the notation of */*, e.g. */ws*. A 3D point represented in Cartesian space is expressed through the vector of the components, e.g. $[x_{P}, y_{P}, z_{P}]$.

### System Calibration

The fiducial localisation error (FLE) allows to measure spatial data points during image guidance [16] and in this work, it was quantified for the stereo endoscope and for the two PSMs.

The FLE is estimated by calculating the average of the measured distance values in terms of the Cartesian position between the *localised* points and the *known* checkerboard dimensions following co-registration. The acquisition of *localised* points will be explained in the experiments section. FLE can be mathematically formulated as follows:

1 $$\begin{aligned} {\text {FLE}} = \frac{1}{n-1} \sum _{i=1}^{n-1} ||p^{l}_{i} - p^{k}_{i}|| \end{aligned}$$

where *n* represents the number of selected points, and $p^{l}_{i}$ and $p^{k}_{i}$ are respectively the Cartesian coordinates of the *localised* and *known* points.

### Rail detection and surface acquisition for kidney registration

The PAF rail has a fiducial represented by a checkerboard composed of squares with a side length of 1 mm used to estimate its pose. The pattern could be easily replaced with any other clinically compatible solution available in the market [17]. The checkerboard tracking and the stereo triangulation functions from MATLAB calibration toolbox were used to determine the location of the rail inside the workspace.
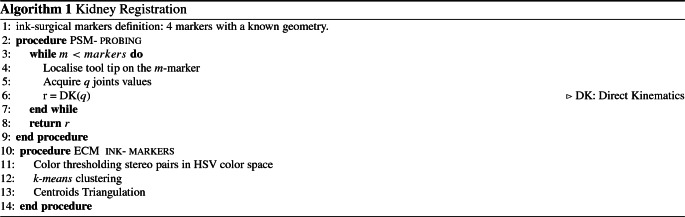


Knowing the rail’s geometrical model, it is possible to compute the location of the grasping site using perspective-n-point (PnP [18]) pose estimation and forward driving the trajectory of the kinematics to grasp.

Registration of kidney soft tissue for image guidance is important since the kidney surface represents the target structure for positioning the image guidance rail. Our experimental set-up follows a previous registration comparison [14] for phantom models adapted to a porcine kidney. Two methods were analysed as shown in the Algorithm 1 formulation: PSM-probing and ECM ink-markers. The PSM-probing procedure returns *r*, which is the probed Cartesian position. The results coming from each method are compared in terms of distances between the real makers representing the ground truth.

### Dynamic time warping trajectory planning and control features

To automatically place the rail on the target position with the robotic tool, two main tasks are needed: generate a trajectory to position the rail and develop a control strategy to optimise the operational performance.

#### Trajectory generation

**Fig. 3:**
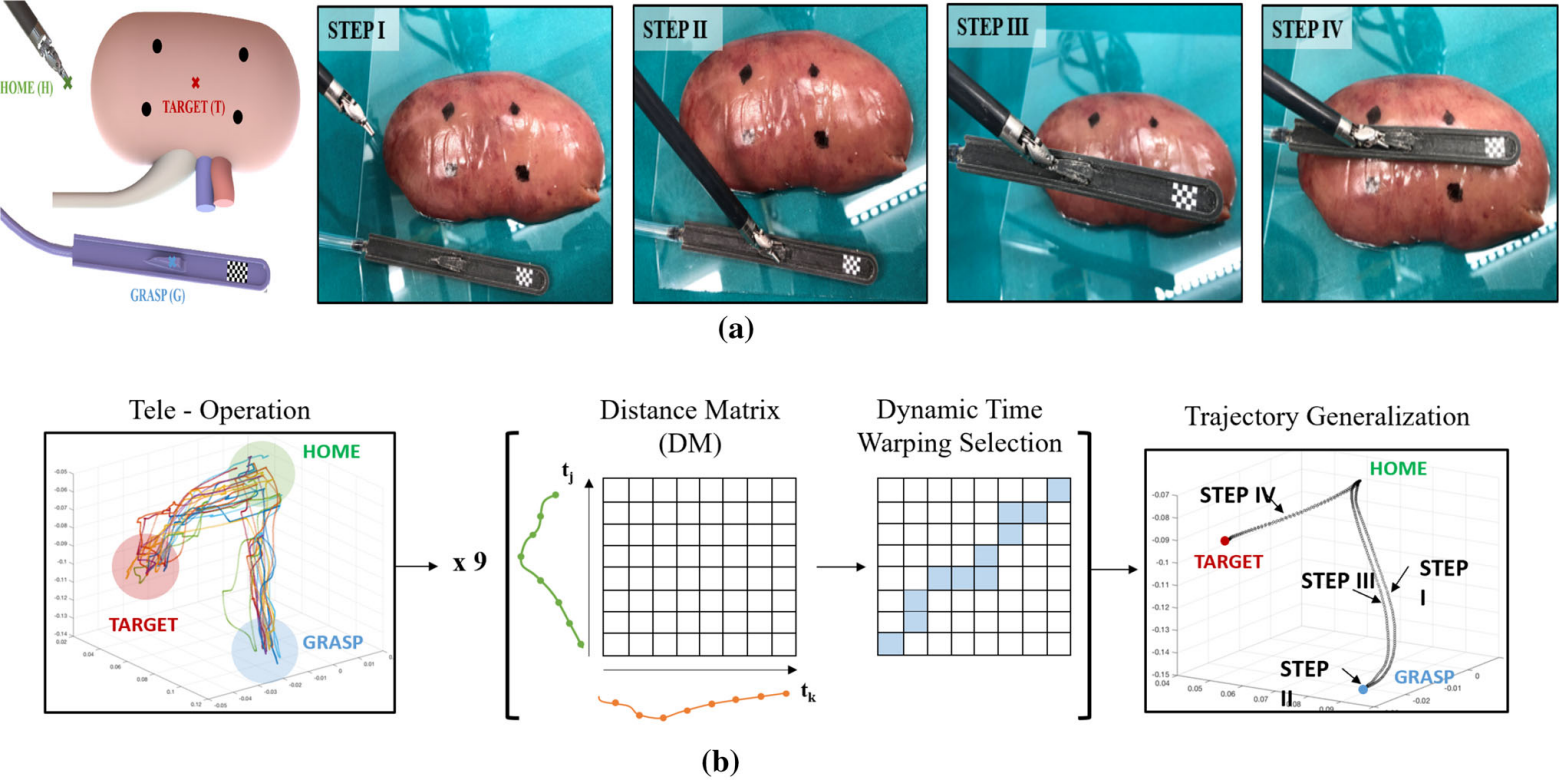
**a** Representation of the different steps of the pick and place task: approaching, grasping, motion and targeting. **b** Block representation of trajectory generation steps. From the left: Cartesian values of tooltip positions, distance matrix considering two trajectories at the time, followed by dynamic time warping selection used to obtain the final trajectory

We separate the pick and place task in four stages which are shown in Fig. 3a.

STEP I, the tooltip starts from the *home* position defined in the 3D space by $[x_{H}, y_{H}, z_{H}]$.

STEP II, the robotic tool approaches the *grasping* site in the central part of the rail. $[x_{G}, y_{G}, z_{G}]$ is the Cartesian position of the grasping point coming from the rail detection through the stereo camera and successively triangulate in the 3D space.

STEP III, the tip moves back to the predefined *home* position.

Finally, in STEP IV, the tool holding the rail moves towards the kidney surface to reach the *target* point $[x_{T}, y_{T}, z_{T}]$. The Cartesian position of the *target* point is computed as the centroid of the bounding box generated by the four ink markers.

Dynamic time warping (DTW) [19] was used to estimate the path followed during the transition phases among the described steps: 10 different repetitions of the same locating task were executed in tele-operation by a trained operator. During these procedures, both the Cartesian and the joints values were acquired using the software framework of the dVRK (Fig.3b—left image). Considering two Cartesian trajectories at the time $t_{j}$ and $t_{k}$, dynamic time warping between them can be formally defined as a minimisation of the cumulative distance over potential path between two time series elements, as shown in the following equation:

2 $$\begin{aligned} {\text {DTW}}(t_{j},t_{k}) = min \Big [ \sum _{i=0}^{P} \delta (w_{i}) \Big ] \end{aligned}$$

where $w_{i}$ indicates a point (j,k) identifying one element from $t_{j}$ and one from $t_{k}$ which are aligned. $w_{i}$ represents each element of the matrix *W* defined as *distance matrix (DM)*. The *DM* has the dimension of the element of $t_{j}$ times the element of $t_{k}$, indicated with *P* in the equation. The values inside each cell of the DM are computed as: $|T_{j}-T_{k}| + min( D(j-1,k-1), D(j-1,k), D(j,k-1) )$ where $T_{j}$ and $T_{k}$ are two respective elements from $t_{j}$ and $t_{k}$ and D($\cdot$,$\cdot$) represents the values of the previous computations. The procedure is then iterative replicate for the 10 trajectories.

During STEP II, it is important to guarantee the correct orientation of the tool in order to achieve a solid grasp. This can be done tuning the last three joints of the PSM arm that are indicated with $[q_{4}, q_{5}, q_{6}]$. The joints values have been filtered and averaged in order to define the final value. To account for uncertainties and minor errors, some other control features were added to enhance the performance of driving the tool. Starting from the estimation of the grasping point: $\mathbf {x_{G}}$ defines the position of the first target motion. This point is reconstructed in 3D space starting from the stereo pairs. In the dVRK, the baseline between the two cameras is only few millimetres and this reflects consistent uncertainty in the depth estimation, which are also enlarged by the small dimensions of the rail’s fiducial. Once the depth component of the *grasping* position is estimated, it is then compared with the respective component of the kidney surface. Since the two objects are located on the same table surface, their depth estimation cannot differ more than the thickness of the kidney itself. This works as a safety initial control to ensure that the rail tracker is working correctly.

#### Control strategy

A further control policy based on visual servoing was added to enhance the performance of manipulating the tool, starting from STEP II. The system does not present any tool tracking node, but once the relationship between the rail and the tool is geometrically established, after the grasping phase, it is possible to infer the same information. The position of the tooltip is extracted dynamically and transposed in the $\textit{/ws}$ and compared to the position acquired through the dVRK and transposed in the same space. This error function is then minimised while proceeding to the next step. During STEP IV, an additional control measure is added in order to be sure that at the end of the task the sucker rail is located parallel to the kidney surface. This control was implemented comparing the known position of the rail and the registered kidney surface at the end of the task.

## Experiments

### Data acquisition for calibration

Transformations between images and the robot coordinates were computed and the accuracy of the tooltips’ position was examined through experiments. Forty-five image pairs of a 7 row by 10 column checkerboard acquired from different endoscopic poses were used as input for the stereo calibration (Fig. 2—Stereo Calibration). Then, the MATLAB toolbox [20] was used for this step, which first solves the intrinsic and extrinsic parameters of the camera in a closed form considering zero lens distortion and as second step estimates all parameters simultaneously including the distortion coefficients using nonlinear least-squares minimisation. Seven additional image pairs of the checkerboard were acquired in order to determine the pose of the left camera inside the workspace. The corner intersections of the checkerboard have been extracted from these frames, and point-registered with the known dimensions [21] (Fig. 2—Left Camera Pose Estimation). For the two PSMs, equipped, respectively, with the LND and the Pro-Grasp, the FLE was characterised carefully probing 20 intersection points in a 3D printed checkerboard (side length 10 mm) for each of the tools (Fig. 2—Workspace Calibration). Every time a point in the grid was touched, the robot encoder’s values were recorded and used in the forward kinematic model of the dVRK to localise the 3D Cartesian position. The points were then projected back using the known transformation, and compared with the checkerboard dimensions, this procedure was repeated for each PSMs. Lastly, the FLE components for the stereo endoscope were taken as a difference between the localised points inside the frame and the known checkerboard dimensions following co-registration.

**Fig. 4:**
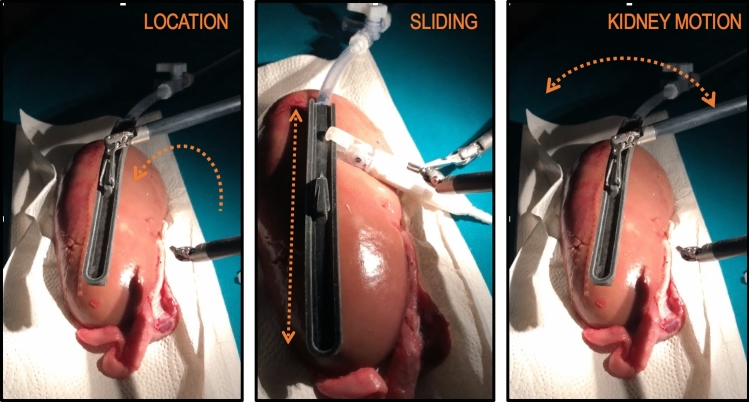
Representation of the three experimental tasks of the pre-clinical study. From left to right: Location task, Sliding task and Kidney Motion task

### Task automation

A preliminary test of the repeatability of the task is run to validate that the kinematics can be used for guiding the robot. The rail is positioned in the field of view of the endoscope and the LND tooltip starts from the *home* position. The task is completed when the tool has grasped the rail and precisely placed it on the kidney surface and headed back to the* home *position. The whole procedure is repeated for 6 times with the same initial conditions. The experiment is defined as follows: the rail is deployed in the field of view of the stereo endoscope without a pre-defined orientation to emulate the clinical protocol. During laparoscopic surgery, external devices are inserted inside the patient, “dropping” them via an auxiliary trocar. If the rail reaches an upside-down position, it is relocated by the assistant using the suction pipe so that the marker is always visible. Once the system is detected inside the endoscope field of view, the robot starts its motion grasping the target and locating it on the organ surface following the pre-planned trajectory. The automated part of the task is considered concluded when the rail is effectively in suction with the kidney itself and the surgeon can start the tele-operated sliding of the probe. A dataset of 40 acquisitions has been recorded to test the overall architecture.

**Fig. 5:**
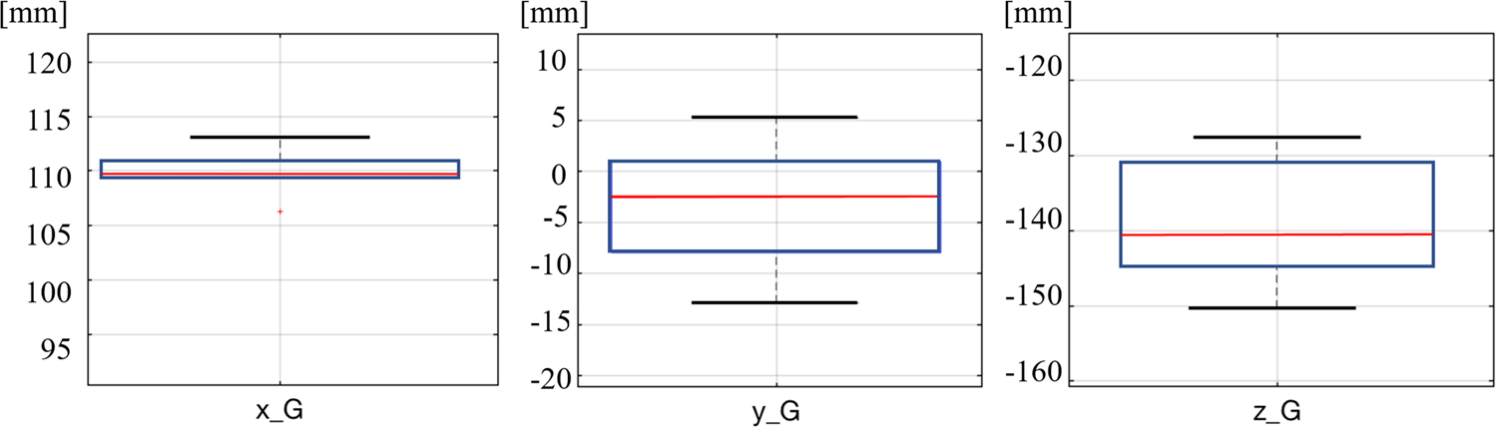
Repeatability test: the box plots show according to the three different axes $(x_G, y_G, z_G)$ the mean values of the grasping point estimate in the 3D space for all the 6 acquisitions

### Pre-clinical user study

Five surgeons took part in the acquisitions, with different years of experience in RAPN. The surgeons were not allowed to familiarise with the system before the testing and no instructions were given on how to execute the task in order to minimise their bias. Furthermore, all the participants conduct the study independently. During the acquisitions, they had to complete three main tasks described as follows: *Location*: the surgeon grasps the rail and place it over the kidney surface, ensuring that the suction line is firmly attached. The *Sliding* task follows: once the rail is in place the surgeon has to pair the probe adaptor with it and complete a full slide back and forth, concluding the task removing the probe from the rail. In the last task, *Kidney Motion*, the rail is grasped while paired with the kidney surface and used to move the kidney with circular movement in respect to the main longitudinal axis as can be visualised in Fig.4 on the right. Each task was repeated three times and the variables measured are: execution time $\mathbf {T_{\text {exe}}}$ in minutes, success rate **SR** represented by a fraction where the denominator shows the number of attempts needed before succeeding the task, difficulty in using the system **DF** scored from 1 to 10, and how much they were willing to use the system in real clinical practice **WU** scored from 1 to 10.

**Fig. 6:**
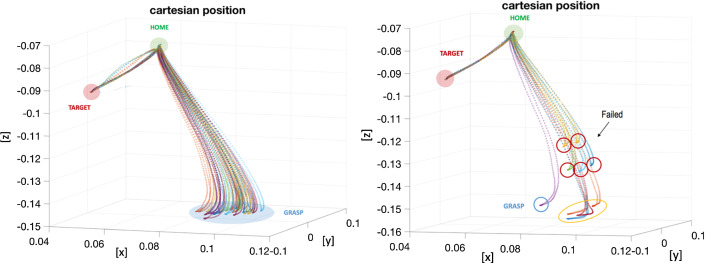
On the left: representation of the Cartesian position of the tooltip in the workspace in metre. Thirty out of 40 acquisitions are represented. All successful and all starting with a different initial position of the rail. On the right: representation of the Cartesian position of the tooltip in the workspace in metre. Ten out of 40 acquisitions are represented. The blue circled one represents a well-executed task plot as a visual reference for the other trajectory. The red circles indicate the 5/40 executions failed, while in the yellow ones the task was properly executed but with a not precise depth estimation

## Results

### Calibration and kidney registration

A quantile–quantile (QQ) plot (Fig. 5) was used to characterise the FLE distribution. The mean and the standard deviation obtained of the FLE magnitudes are the following: for PSM1 1.10 ± 0.58 mm, PSM2 4.33 ± 0.78 mm and ECM 0.93 ± 0.56 mm. Notably, the value related to the PSM2 equipped with the Pro-Grasp is significantly higher than the one with the LND. During the probing procedure, the nominal DH parameters provided with the intuitive surgical API were used for both the robotic tools. In the case of the Pro-Grasp, the probing procedure results are inaccurate due to the hardware design. The rounded shape of the Pro-Grasp tip makes difficult to isolate the same precise point to guarantee a repetitive probing, while the design of the LND allows for more exact and accurate acquisitions. Based on these outcome values, it has been decided to use the LND as grasping tool during the experimental acquisition instead of the Pro-Grasp, although the rail has been designed for that particular tool.

Regarding the kidney registration, the accuracy is quantified by the error in the markers reconstruction for each method in terms of Euclidean distance between the markers themselves. Given the ground truth of 50 mm, the value obtained from surface tracking with the PSM tip is 51.23 ± 0.44 mm, while with the ECM triangulation of surgical ink markers is 54.10 ± 0.88 mm. Unsurprisingly, given the small baseline 5.4 mm of the stereo camera in the da Vinci endoscope, localisation registration with PSMs was more accurate than with endoscope-based technique. These results do not affect the experiment negatively since probing techniques can be potentially computed in real surgical environments.

**Table 1:** The values represent the average values among the three repetitions for each task

	Surgeons ID	Location				Sliding				Kidney Motion			
		$\mathbf {T_{\text {exe}}}$	SR	DF	WU	$T_{\text {exe}}$	SR	DF	WU	$T_{\text {exe}}$	SR	DF	WU
S1	Urology 4 y.o.e.	1	1/1	1	1	5	1/6	7	0	1	1/1	1	8
S2	Urology 1 y.o.e.	1.5	1/5	3	0	6.3	1/7	6	0	1.2	1/2	2	4
S3	Urology 3 y.o.e.	3	1/3	5	5	8	1/8	8	5	1.4	1/2	2	8
S4	Urology 2 y.o.e.	2.5	1/4	4	4	7.1	1/5	7	4	1.1	1/1	3	7
S5	Urology 2 y.o.e.	4	1/3	3	3	6.6	1/5	7	4	1.2	1/2	2	6

y.o.e stays for “years of experience” in robotic surgical operation. The execution time is reported in minutes

### Automation results

Results from the repeatability test are shown in Fig 5. The box plots show the mean values of the position estimated during the acquisition, according to the three Cartesian axes in the */ws*. Although the starting location of the rail was always the same, due to errors coming mainly from the triangulation, the Cartesian position of the grasping point slightly varies among the 6 repetitions. The values related to the z-axis are greater than the other two axes, confirming that the main error component is due to depth estimation. Those values are still small enough to guarantee a correct grasp in all the repetitions since the length of the tool gripper jaws can compensate them. Based on these results, we decided to proceed on building the architecture on the visual feedback coming from the endoscope instead of using external cameras with increased baseline. This would improve the accuracy of the results but move us a step further away from an environment more similar to the surgical one. Fig. 6 left side shows the results coming from the experiments. The success rate is of 35 acquisitions over 40, while in the remaining 5/40 the tooltip is not able to reach the rail. These failed executions can be attributed to the inaccuracy associated with the tracking and reconstruction of the rail in the 3D space. Additionally, in 4/40 acquisitions the task was correctly completed but with some clear error in the pose estimation of the rail (highlighted with the yellow circle in the Fig. 6 on the right side). As a matter of fact, the reconstructed position appears to be on the correct plane but parallel compared to the real one. When the tool tries to grasp the rail, it generates a small sliding movement on the plane, due to the fact that the reconstructed position appears to be further down compared to the robot reference frame. In this case, using external cameras with increased baseline would improve the success rate of this experiment. The average time among all the acquisitions to execute the autonomous part was *42 seconds*.

### User study results

Table 1 reports the results obtained from the surgeons during the tests. Values are shown as the mean values among the three different repetitions of the same task. The different surgeons are represented by “**S**” followed by a number. Comparing the execution times between the automated task and the one executed by surgeons, it is possible to see how $\mathbf {T_{\text {exe}}}$ increases by an average of 85 ± 109 s among all the executions.

## Discussion and conclusion

In this paper, we have reported preliminary experiments showing that automatic positioning of a PAF rail is possible by combining motion planning and visual servoing. Our framework can be used to place the PAF rail onto tissue by autonomous instrument motion following a planned trajectory and subsequently the rail can be used to manoeuvre a US probe. We implemented and compared calibration accuracy of this approach with two different dVRK instruments. Experimentally our pre-clinical case study showed surgeons’ interacting with automation of procedural sub-tasks. Results highlighted the need to build inherent user flexibility and make the system compatible with every tool, not only the LND and Pro-Grasp, meaning that design process for the rail system and handles can be improved. The proposed solution, although in a preliminary stage, showed promising results in terms of execution time. Multiple difficult challenges remain for translating such technology within more realistic experiments and a clinical environment. Examples include robust vision algorithm for taking into account tissue deformation or coping with dynamic effects like bleeding that obscures information inference for visual servoing. In addition to the positioning of the PAF rail, automation of the US probe manipulation requires additional motion planning and adaptation to tissue geometry. Further work is also needed for adaptive control in the presence of physiological motion and more comprehensive clinical workflow studies are necessary including the use of a functional US probe. These are some of the aspects ascribable to the failed executions highlighted in Fig. 6. We believe that the introduction of the described technical features in the experiment will improve the success rate of the experiments allowing to meet the clinical standards. Although some of the technical aspects related to the problem still need to be addressed as stated above, the authors believe that the adoption of this new device and the introduction of a new clinical protocol are fundamental to boost towards partial automation in RAPN.
